# Evaluating Suicidal Risk in GLP-1RA Therapy: An Umbrella Review of Meta-Analytic Evidence

**DOI:** 10.3390/healthcare13222958

**Published:** 2025-11-18

**Authors:** Cristina Ștefănescu, Elena Alexandra Bratu, Ana Maria Pelin, Denisa Boroi, Bianca Daniela Crecan-Suciu, Victorița Ștefănescu

**Affiliations:** 1 Faculty of Medicine and Pharmacy, Doctoral School, “Dunarea de Jos” University, 800008 Galati, Romania; cristina.stefanescu@ugal.ro (C.Ș.); victorita.stefanescu@ugal.ro (V.Ș.); 2 Faculty of Psychology and Educational Sciences, University of Bucharest, 050663 Bucharest, Romania; denisa.boroi@yahoo.com; 3 Department of Neurosciences, “Iuliu Hatieganu” University of Medicine and Pharmacy, 400012 Cluj-Napoca, Romania; suciu.bianca@umfcluj.ro

**Keywords:** glucagon-like peptide-1 receptor agonists, GLP-1RAs, type 2 diabetes, obesity, suicidal ideation, suicidal attempts, self-harm behaviors

## Abstract

Objective: This is the first umbrella review to integrate the available research syntheses on the associations between glucagon-like peptide-1 receptor agonists (GLP-1RAs) and suicidality. Methods: A systematic search within the main databases (MedLine, PubMed, PsychInfo, Web of Science, Science Direct, Sage, Wiley, Scopus, Google Scholar, Proquest, and Cochrane Library) was performed using the main key words. Out of a total of 50 initial studies, a final number of 12 reviews and meta-analyses were included in the study. Results: Except for two observational studies, the results consistently found no significant association between GLP-1RAs and suicidality, though improvements in symptomatology (as found in five studies), quality of life (three studies), and diabetes distress (one study) were highlighted. Although no causal effect could be yielded, an association was observed between suicidality and having unrealistic treatment expectations, being underweighted, and being treated with semaglutide and liraglutide (as indicated by two studies). Conclusion: GLP-1RA treatment represents a safe option when treating diabetes/obesity. However, a careful assessment of treatment expectations and of suicide risk is needed in order to attenuate potential suicidal tendencies that already exist.

## 1. Introduction

The global prevalence of metabolic disorders is escalating. By 2035, over 1.3 billion individuals will have diabetes—predominantly type 2 diabetes mellitus (T2DM)—while more than 50% of the global population may be overweight or obese [1,2]. This alarming trend highlights the urgent need for therapeutic interventions that address both glycemic control and sustainable weight loss. Glucagon-like peptide-1 receptor agonists (GLP-1RAs), originally developed for T2DM management, have emerged as promising dual-purpose agents targeting both metabolic and weight-related outcomes [3,4]. This is due to GLP-1RAs’ capacity to activate the GLP-1 hormone (which stimulates glucose-dependent insulin secretion, suppresses glucagon release, and delays gastric emptying) and to modulate the brain regions involved in appetite control (thus acting on the release of neurotransmitters and peptides involved in hunger and energy regulation) [5]. These mechanisms contribute to weight loss and may also modulate neuroendocrine and reward circuits implicated in mood regulation [5].

GLP-1RAs—including liraglutide, semaglutide, and the dual agonist tirzepatide—have seen expanded regulatory approval. Liraglutide was approved for T2DM in 2010 and obesity in 2014; semaglutide followed, with T2DM approval in 2017 and weight-management approval in 2021 [6,7]. Tirzepatide, targeting both GLP1 and glucose-dependent insulinotropic polypeptide (GIP) receptors, gained T2DM approval in 2022 and is currently undergoing evaluation for obesity indications [6]. These approvals reflect the robust evidence supporting GLP-1RAs’ effects on appetite, gastric motility, and energy homeostasis [7,8].

Clinical trials have demonstrated that once weekly semaglutide (2.4 mg) induces substantial weight loss when combined with lifestyle intervention [9,10]. Nevertheless, emerging post-marketing pharmacovigilance data raise concerns regarding potential neuropsychiatric adverse events (AEs). A comprehensive analysis of the FDA Adverse Event Reporting System (FAERS) identified over 25,000 neuropsychiatric incidents, including headache, insomnia, anxiety, sensory disturbances, and suicidal ideation, with semaglutide signals notably prominent (reporting odds ratio [ROR] 2.55 for suicide ideation in obesity populations) [11,12].

These signals have prompted safety reviews by regulatory agencies. The European Medicines Agency (EMA) initiated formal evaluations of semaglutide and liraglutide in 2023 due to reports of suicidal behavior and self-harm among users [13]. Meta-analyses of randomized controlled trials (RCTs), however, generally show no significant increase in psychiatric AEs, though these often exclude high-risk individuals and may be too underpowered to detect rare psychiatric events [14,15,16]. For example, a meta-analysis of 31 RCTs (*n* ≈ 85,000) found no significant difference in psychiatric disorders (OR 0.97; 95% CI 0.83–1.15) or suicidal behavior (OR 0.86; 95% CI 0.47–1.56) between GLP-1RAs and comparators [14]. Likewise, AHA Circulation’s pooled analysis of 36,000+ subjects yielded an OR of 0.88 (95% CI 0.60–1.29) for suicide or self-harm [17].

Beyond safety concerns, there is growing interest in GLP-1RAs’ therapeutic potential in mental health. A meta-analysis of 2071 participants revealed modest but significant reductions in depressive symptomatology (SMD −0.12; 95% CI −0.21 to −0.03) across various GLP-1RA treatments [18]. Preclinical and genetic studies link GLP1R activation to anti-inflammatory, and neurotrophic and neuroplastic effects, benefiting mood, anxiety, and reward-related disorders [19,20]. Mendelian randomization studies suggest that genetic proxies for GLP1R activation may lower risks of schizophrenia (OR 0.72), bipolar disorder (OR 0.91), PTSD, and autism, though there may be increased risk for obsessive–compulsive disorder (OCD) [20].

Conversely, several large observational datasets have flagged increased neuropsychiatric risks. A TriNetX cohort (*n* ≈ 162,000) reported a 98% increased risk of psychiatric disorders, including major depression (HR ~2.95), anxiety (~2.08), and suicidal behavior (~2.06) in GLP-1RA users; however, confounding via indication and population heterogeneity limit causal inference [21]. FDA FAERS data also highlight initial signals of suicidality but are subject to reporting biases and lack denominator data [20].

Individual vulnerability factors—such as age, sex, psychiatric history, dosage, and treatment indication—may moderate neuropsychiatric risk. For instance, psychiatric symptoms appear more prevalent in semaglutide users undergoing high-dose regimens for obesity than for diabetes [8,13]. The rapid weight loss itself, with concomitant neurochemical shifts and psychosocial stressors, likely contributes to these effects [14,15,16].

Collectively, this evidence underscores the need for rigorous post-marketing surveillance, stratified psychiatric risk assessment, and the integration of mental health monitoring with weight-loss interventions. Clinicians must remain alert to both adverse psychiatric outcomes and potential mental health benefits when prescribing GLP-1RAs, ensuring informed, patient-centered treatment decisions.

To our knowledge, despite the increasing number of synthesizing studies on the link between GLP-1RAs and suicidal behaviors, no umbrella review has been performed. This is the first study to synthesize the current state of the literature regarding the relationship between GLP-1RAs and suicidality (defined as any of the following elements: suicidal ideation, self-harm, and suicide attempts). As such, this umbrella review addresses the following research question, framed by the PICO model:

Population (P): Adults with type 2 diabetes or obesity;

Intervention (I): Treatment with GLP-1 receptor agonists;

Comparator (C): Placebo, or other antidiabetic or anti-obesity medications, or no treatment;

Outcome (O): Incidence of suicidality, including suicidal ideation, suicide attempts, and self-harm behaviors.

Accordingly, this study aims to determine whether GLP-1RAs are associated with increased suicidality and to synthesize the psychiatric and quality-of-life outcomes reported across systematic reviews and meta-analyses.

## 2. Materials and Methods

### 2.1. Literature Search Procedures

The systematic literature search was conducted between the months of June and July 2025 and included the following major databases: MedLine, PubMed, PsychInfo, Web of Science, Science Direct, Sage, Wiley, Scopus, Google Scholar, Proquest, and Cochrane Library.

To identify potential relevant studies, we searched for studies using the following search string: (glucagon-like peptide-1 receptor agonist OR GLP-1 receptor agonist) AND (suicide OR self-injury OR self-harm OR suicidal ideation OR suicidal behavior OR suicide attempt OR suicide risk) AND (diabetes) AND (review OR meta-analysis). The search string was used in the same order for all databases. The search covered all available publication years from database inception to July 2025, and no language filter was applied. More details can be found in Appendix A.

### 2.2. Inclusion Criteria

We sought to include studies that (a) documented the link between GLP-1RAs and suicidality (meaning suicidal ideation and/or suicide/self-harm attempts); (b) were synthesis studies (systematic reviews, qualitative/narrative reviews, scoping reviews, or meta-analyses); (c) were performed on adults and/or children; (d) were based on samples diagnosed with diabetes or obesity; and (e) addressed suicide ideation or behavior either as a main outcome or as a secondary one. No language filter was employed. For studies written in any language other than English, the text was translated into English with the help of ChatGPT [22], then checked for accuracy by one author who is fluent in Spanish (the language the article was written in; this applied only for one study, as all the other studies were written in English). Eligible studies that could not be accessed in full-text form were also included (although they could not be analyzed completely), since they could still offer relevant information regarding the main topic.

Studies of any design other than a systematic/qualitative review or meta-analysis, as well as studies that did not address suicide ideation or behavior, were excluded.

### 2.3. Data Extraction and Quality Assessment

Based on an adapted version of the JBI Data Extraction Form for Systematic Reviews and Research Syntheses [21], we extracted data regarding the study type, target population, and psychological condition addressed, as well as the duration of GLP-1RA treatment. We also collected information on reported effects on suicidality (including effect sizes and heterogeneity for meta-analyses), other adverse effects, and additional psychological outcomes, such as quality of life. Further methodological details were recorded, including the number of databases searched, study quality assessments (when available), and the total number of included studies [23]. Finally, demographic characteristics were extracted, such as total sample size, mean age, gender distribution, and comorbid diagnoses.

The quality of the included studies was assessed by using the JBI Checklist for Systematic Reviews and Research Syntheses [21], which consists of 11 questions addressing aspects such as the appropriateness of the search strategy, appropriateness of the methods used to combine studies, etc., with four types of responses (“yes”, “no”, “not reported”, and “not applicable”). This tool was used based on the utility of the questions and on the fact that it is one of the gold-standard instruments used for the quality assessment of studies [20]. Discrepancies were resolved by discussion to reach consensus, then a third author was consulted for feedback.

### 2.4. Pre-Registration

This study was prospectively registered on the PROSPERO platform, number 3949159.

## 3. Results

### 3.1. Selection of Studies

**Figure 1:**
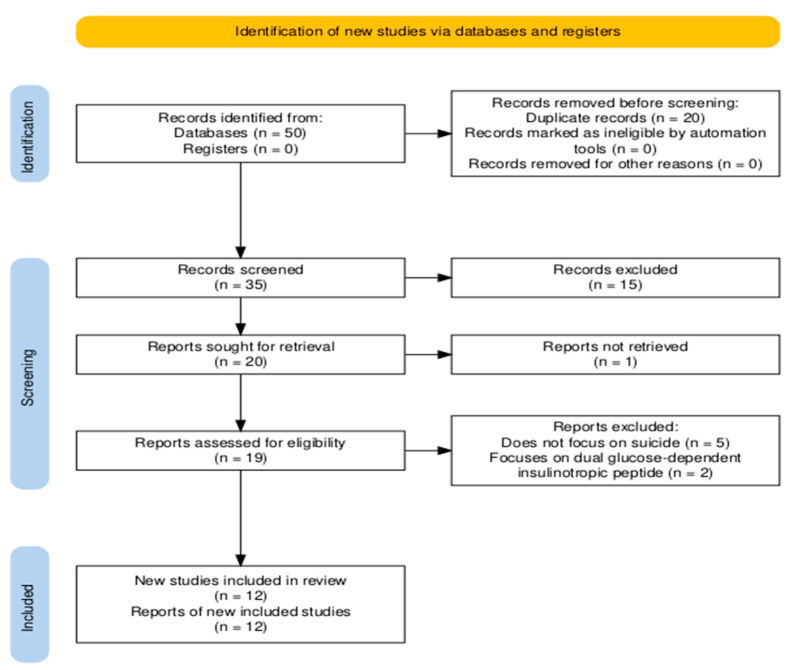
The selection process.

The main search found a total of 50 studies, 35 after duplicates’ removal, which were screened based on the abstract (see Figure 1 for more details about the selection process).

Next, 15 studies were excluded (for not meeting the inclusion criteria) and 19 studies were assessed in full-text form, as one study could not be found and offered very little information regarding suicide, in order to decide on the appropriateness of their inclusion. In the end, twelve studies were included in the analysis, as seven studies did not meet the criteria (five did not focus on suicide, whereas two focused on another type of medication). In order to synthesize all the available results, two studies were included only in the abstract format, due to the unavailability of the full-text format. The selection process was performed independently by two authors, who discussed any discrepancies in the selection. Also, the whole process was performed based on The Preferred Reporting Items for Systematic Reviews and Meta-Analyses (PRISMA) flow diagram [24], which was generated with the help of Shiny App Tool [24].

### 3.2. Study Characteristics

In total, 12 studies were included in the analysis. This number covered four systematic reviews, two meta-analyses, four systematic reviews and meta-analyses combined, one scoping review, and one narrative review, which were published between 2023 and 2025. The number of the included studies relevant to suicide varied from 2 to 36, and assessed studies employing the following various types of methodological approaches to the topic: randomized control trials (RCTs), observational studies, post hoc analyses, case series, and pharmacovigilance studies. The sample sizes ranged from 6 to 131,255,418 participants, covering both children and adults with ages from 0 to 85 years old (although only two studies, of those that reported age, included children) from various countries, and there was a slight prevalence of males (53%). All participants were diagnosed with type 2 diabetes and/or obesity and a significant proportion of studies also included participants diagnosed with several psychological conditions (such as depression, schizoaffective disorders, substance use, obstructive sleep apnea, etc.). More details about study characteristics can be found in Table 1.

Search strategies varied across reviews, with the number of databases searched ranging from two to eight. The most frequently used databases were PubMed/MEDLINE, PsycINFO, and Web of Science. Quality appraisal of the primary studies was reported in approximately two-thirds of the reviews. Those based primarily on randomized controlled trials generally indicated a low risk of bias and no evidence of publication bias [8,17], whereas reviews incorporating observational or pharmacovigilance data reported more heterogeneous or insufficiently detailed assessments of study quality (varying from high to moderate quality in those reporting the results of this assessment) [25,26].

**Table 1:** Study characteristics.

Study	Psychological Disorder	Study Type	Type of the Included Studies	N of Studies	N Databases	Quality of Studies	N Sample	Mean Age	Gender Prevalence	Sample
Barroso [27]	MDD	systematic review	RCTs and prospective cohort studies	7	3 (PubMed, Google Académico, Cochrane)	No	no synthesized details regarding study characteristics	N/R	N/R	patients with type 2 diabetes
Breit [28]	mental health	systematic review	RCTs (17), CTs (clinical trials, 2), prospective observational studies (7), retrospective (4), retrospective case series (1), two open-label studies (2), and post hoc pooled analyses (3).	36	2 (PubMed, Cochrane Library)	No	samples range: 6–5325 participants	N/R	N/R	overweight patients (all studies), 18 studies on mental illness (schizoaffective disorders bipolar, depression, or the whole spectrum of disorders), and 21 studies on patients with prediabetes or type 2 diabetes
Bushi [29]	suicide risk (suicidal ideation, suicide attempts, suicidal behaviors, and self-injury)	systematic review and meta-analysis	observational cohort (10) and case-control (1) studies	11	3 (PubMed, Embase, Web of Science)	although a quality assessment was performed, no results were reported.	samples range: 6–5325 participants	range from 46.6 to 65.9 years old	N/R	ranged from type 2 diabetes patients, to overweight/ obese adults, to general users of GLP-1RAs.
Di Stefano [30]	suicidality	systematic review	observational cohorts (5), RCTs (2), pharmacovigilance disproportionality analyses (8), post hoc RCT analysis (1)	16	3 (MEDLINE, Embase, APA PsycInfo)	both observational and randomized studies were assessed as having a low risk of bias	N/R	>12 years old	N/R	patients with T2D or obesity
Dutta [31]	depression and suicidality (as secondary outcome)	systematic review and meta-analysis	RCTs	2 studies evaluating suicide risk	7 (Medline, Embase, Cochrane Central, ctri.nic.in, clinicaltrials.gov, global health, and Google Scholar)	low risk of bias	437	N/R	N/R	patients with sleep obstruction apnoea
Ebrahimi [8]	suicide and self-harm	systematic review and meta-analysis	RCTs	27	4 (MEDLINE, Embase, ClinicalTrials.gov, Cochrane)	Most were low risk; 5 high risk due to >5% attrition; and no publication bias detected	59,403 participants (32,357 GLP-1; 27,046 placebo)	59.5 years old	56% males, 44% females	patients with diabetes or obseity
Pierret [9]	serious psychiatric disorders (major depression, suicidality, or psychosis), non-serious psychiatric disorders (anxiety or insomnia).	systematic review and meta-analysis	RCTs (Double-blind placebo-controlled trials)	80 (only 3 assessing suicidality alone)	4 (MEDLINE, Embase, PsycINFO, CENTRAL)	low risk of bias in 99% of studies	107,860	60.1 years old	40.1% females, 59.1% males	patients with diabetes or obseity
Silverii [17]	any psychiatric disorder or suicidal behaviors	meta-analysis	RCTs	31	4 (Medline, Embase, Clinicaltrials.gov and Cochrane CENTRAL Database)	no evidence of publication bias	84,713	-	-	patients with diabetes or obseity
Strumila [26]	suicidal ideation and behaviors	narrative review	discussed studies included commentary, pharmacovigilance data (FAERS, EMA reports), real-world observational studies, and analogies with other interventions	N/A	N/A	N/A	N/A	N/A	N/A	obesity and diabetes
Valenta [10]	depression, anxiety, suicidality, alcohol and substance use disorders, binge eating disorder, psychosis, and autism spectrum disorders	scoping review	animal experiments, RCTs, cross-sectional and cohort studies, pharmacovigilance reports, and case reports/series	81 total (51 animal, 30 human)	2 (MEDLINE, Web of Science)	high quality	N/R	N/R	N/R	diabetes, obesity, psychiatric disorders (depression, anxiety, substance use, binge eating, psychosis, and autism) for human studies
Valentino [32]	suicidality	systematic review	pharmacovigilance and cohort studies	22 (10 pharmacovigilance,12 cohort)	8 (Pubmed, Medline, Cochrane Library, PsychInfo, Embase, Scopus, and Web of Science, Google Scholar)	cohort studies of moderate quality; pharmacovigilance studies were not assessed for quality	samples ranging from 204 to 131,255,418	ranging from 0 to 85 years old	ranging from 53.18% to 65% females	N/R
Wei [25]	suicidality	meta-analysis	observational	5	3 (PubMed, Embase, Scopus)	N/R	746,306	N/R	N/R	diabetes and obesity

Note: N studies = total number of included studies; N databases = total number of searched databases; N sample = number of participants within the analysis; MDD = major depressive disorder; RCT = randomized control trial; CT = control trial; N/R = not reported; N/A = not applicable.

### 3.3. GLP-1RAs and Suicidality

Overall, in studies reporting this aspect [8,9,13,28], treatment duration varied from 4 weeks to 3 years (although most of them had a mean duration of around 7 months; for a more detailed approach, see Table 2).

The most consistent pattern was that there were no effects of GLP-1RAs on any aspect of suicidality. This means there are no higher risks of suicidal ideation, behavior, or attempts in patients using GLP-1RAs than in those who are not treated with this type of medication. This trend held true for both patients with or without a psychological disorder [33]. Furthermore, although limited by the high heterogeneity, one study found a smaller risk of suicide in patients treated with GLP-1RAs [26]. Additionally, quantitative data confirmed this result. Thus, across the six studies providing quantitative reports, the risk ratio for suicidality ranged from 0.56 to 0.83, without a significance level (*p* varying from 0.06 to 0.82 in some cases), but with varying levels of heterogeneity (from 0.00% without significance, to 98%, with a significant impact, *p* < 0.01).

Still, despite this clear trend, two studies composed of observational data found suicidal ideation to be present (not increased) in patients taking GLP-1RAs [34], and self-harm behaviors in underweight patients [35], although a causal relationship cannot be inferred. At the same time, two meta-analyses showed a more nuanced pattern, based on the methodology of the studies and of the GLP-1RAs’ type [27], and based on patient expectations [31]. Firstly, mixed results were found in terms of pharmacovigilance, meaning an increased risk was observed for semaglutide and liraglutide, whereas for the rest of the GLP-1RAs this trend was not supported [27]. Secondly, unrealistic expectations and treatments’ failure to meet these expectations were associated with an increased risk of suicide attempts, while GLP-1RAs alone did not have a direct link to the attempts [31]. Thus, these results indicate the answer may not be so straightforward, which further highlights the need to take more details into consideration.

### 3.4. GLP-1RAs and Other Psychological Effects

Beyond suicidality, several reviews evaluated the broader psychological effects of GLP-1RAs (Table 2). Generally, evidence revealed no worsening of psychological symptoms during treatment and, in some cases, it promoted modest improvements. These improvements were related to depressive symptoms [10,27], alcohol use [10,28], well-being [28], life quality [9,10,29], emotional eating [10], cognition [10], substance use [10,13,26], treatment satisfaction [28], diabetes distress [28], and anxiety [10,26] (of note here is that despite the beneficial effects in studies performed on humans, in animal models some anxiogenic responses were found [34]). Notably, none of the reviews found consistent evidence of increased psychiatric admissions or functional deterioration in populations with mental illness, thus supporting that GLP-1RAs are psychiatrically safe, with possible secondary benefits on mood, substance use, and quality of life, though these effects are generally small and not uniformly replicated.

### 3.5. Quality Assessment of the Reviews

Overall, the quality of the included reviews provided sufficient robustness in order for the results to be taken into consideration (although caution is needed when interpreting the results), as most reviews had a moderate level of quality (see Table 3). The inter-rater agreement on the quality assessment was high (quadratic-weighted Cohen’s κ = 0.88), indicating almost perfect consistency between reviewers. The unweighted Cohen’s κ was 0.82, also indicating substantial agreement. Discrepancies were resolved by discussion to reach consensus, then a third author was consulted for feedback.

Thus, out of the nine reviews included in the JBI quality assessment, three reviews (33%) met the criteria for high quality, five for moderate quality (56%), and one for low quality (11%; due to several methodological issues). Of note, only studies accessed in full-text form were taken into consideration for the assessment, as lack of sufficient information made such an assessment impossible.

Overall, current evidence suggests no consistent link between GLP-1RA treatment and suicidality, although study heterogeneity and methodological variability remain substantial [26].

## 4. Discussion

In this umbrella review, we systematically analyzed 12 research synthesis documenting the link between GLP-1RAs treatment and suicidality in diabetes and/or overweight patients. Overall, the findings did not indicate a consistent increase in the risk of suicidal ideation, suicide attempts, or self-harm behaviors, which may confirm the relative safety of GLP-1RAs (also found in previous studies, such as [27,29,36]). However, given the substantial heterogeneity observed across studies, these results should be interpreted with caution and careful risk assessment is recommended when prescribing this type of medication [8]. One potential explanation for the lack of association is the mechanism of action of GLP-1RAs, which primarily target brain regions involved in appetite and reward regulation rather than mood regulation [8], suggesting no clear biological pathway linking these medications directly to changes in suicidality. Although the primary neurobiological actions of GLP-1RAs involve appetite and reward regulation, recent neuroimaging and molecular studies indicate that GLP-1 receptors are also expressed in regions implicated in mood and anxiety regulation, such as the prefrontal cortex, hippocampus, and amygdala. This distribution suggests potential indirect effects on emotional and impulse control processes. However, current clinical evidence does not support a causal association between GLP-1RA use and suicidality. Future research integrating neuroendocrine and affective neuroscience perspectives may clarify this pathway.

Nonetheless, our synthesis indicates that certain factors, such as unrealistic treatment expectations or being underweight, could be associated with a heightened risk of suicidal thoughts and behaviors [30]. These risks may arise from disappointment due to unmet expectations or the challenges of adapting to rapid changes in body weight. Notably, some evidence suggests that semaglutide and liraglutide, which are associated with faster and more pronounced weight loss compared to older GLP-1RAs, may be linked to an increased emotional burden and potential suicidality [27,30].

On the other hand, the beneficial effect on other psychological aspects (ameliorated depressive symptoms, greater well-being and quality of life) could be present due to the improvements in diabetes/obesity treatments, which are associated with more positive emotions and better physical functioning [28]. These benefits may also be due to the effect GLP-1RAs have on the brain reward area and on the neuroplasticity process [28].

However, caution is recommended when interpreting these results, as several limitations emerge. Firstly, there is a possibility that some studies may have been analyzed in different meta-analyses/systematic reviews. This means a higher risk of some results being overstated and others being underestimated due to the lack of representation [37]. Despite this issue, given the consistency of the findings across the various methodologies of the studies (both prospective and retrospective), it is unlikely that results would have been significantly affected. Future umbrella reviews should take into consideration the use of several tools to quantify the studies’ overlaps. This could ensure a higher level of reliability in the results, as well as a more nuanced interpretation of the obtained data. Secondly, a high degree of heterogeneity was systematically found within the included studies, and this could have stemmed from the diversity of the included populations, treatments or methodology, thus reducing the reliability of the effects. While we were unable to formally explore the sources of heterogeneity at the primary study level, we emphasized the consistency of direction and the overall trends across reviews rather than the absolute magnitude of pooled effects. Future umbrella reviews should consider stratified analyses or re-analyses of primary trial data to better account for these variations. Lastly, taking into consideration the lack of available information regarding various details (e.g., previous suicidal ideation/behavior, duration of treatment, GLP-1RAs types, measurements of treatment expectations, stratified data on different age intervals, details regarding the socioeconomic status of the sample, etc.), we were unable to provide a more complex picture of the investigated relationship. This is especially of note as some primary studies highlighted the presence of some nuances (such as the risk of suicide being present in patients with unrealistic expectations or in patients treated with semaglutide and liraglutide [8,14,32]), which may explain the positive results regarding suicidal behaviors found in some primary studies [16,27]. In the future, primary studies should focus on the link between suicidality and treatment expectations, or on the individual impact of specific types of GLP-1RAs, alongside other possible confounding factors (such as optimism, social support, or other socio-economic factors, which can act as risk factors for suicide—e.g., reduced financial resources and stressful life events [31]. Mechanistic studies integrating neuroimaging and endocrinological biomarkers may also elucidate the neurobiological pathways linking GLP-1 signaling with affective regulation and suicidality.

## 5. Conclusions

All in all, this umbrella review reiterates the safe use of GLP-1RAs in patients with diabetes and/or obesity with respect to suicidal ideation/behaviors, and offers a more nuanced view on the topic. Thus, prescribing GLP-1RAs remains a viable option in treating diabetes/obesity, even resulting in several improvements in life quality [25,28,31], levels of psychopathology [28,30,34], or diabetes distress [28] in some cases, which further supports the treatment efficacy. Our results also emphasize the need for the careful assessment of patients’ treatment expectations, as an increased tendency in suicidality was found after failures in meeting these expectations [31]. Furthermore, thorough monitorization should be implemented for patients with high levels of risk, as well as in patients treated with semaglutide and liraglutide [27] (although a casual relation was not found), potentially due to the rapid drops in weight loss [31,38].

## Figures and Tables

**Table 2:** Results regarding treatment and its effects.

Study	Treatment Duration	Reported Adverse Effects	Results Regarding Suicide	Other Psychological Effects	Effect Size (For Meta-Analyses)
Barroso [27]	N/R	No	No causal effects on suicide behaviors were found.	Decreases in depressive symptoms were found.	N/A
Breit [28]	4 weeks- 3 years	No	Mental illness samples: No effect on suicidality symptoms (four studies). Samples without mental illness: No effect on suicidality (one study, no other stud investigated suicidality).	Mental illness samples: No significant increase in psychiatric admissions (five studies), no significant effects on quality of life or functioning (two studies), improved psychopathology (two studies), no worsening effects on psychopathology (four studies), or reduction in alcohol use (two studies). Samples without mental illness: No effect on psychopathology (three studies), improved well-being (three studies), mental health (two studies), quality of life (eight studies), glycaemic control (one study), weight loss (two studies), treatment satisfaction (two studies), or reduced diabetes distress (two study).	N/A
Bushi [29]	N/R	No	The narrative analyses found that compared to other medications (eg., DPP-4 inhibitors), there was a lower risk of suicide attempts (three studies), no significant effects on suicidality (four studies), or no increase in suicidal ideation (one study). Compared with non-GLP-1RAs, results showed a lower risk of suicide attempts (one study) or no effect on suicidality (three studies).	N/R	A risk ratio quotient was pooled based on four studies. No significant results in suicidal outcomes between GLP-1RAs and other drug classes (RiskRatio = 0.568, 95% CI: 0.077–4.205); substantial uncertainty in the true effect (prediction interval, 0.001–218.938); high-heterogeneity (I^2^ = 98%, *p* < 0.01).
Di Stefano [30]	N/R	-	No effect on increasing suicide behavior. Although pharmacovigilance studies showed mixed effects (higher reporting rates compared to other antihyperglycemic agents), there was no causal effect.	N/R	N/A
Dutta [31]	N/R	-	Although the risk ratio analyses indicated a reduced tendency in suicidality for the experimental group, the effect was not significant	N/R	RiskRatio = 0.76 (95% CI: 0.07–7.96); *p* = 0.82; I2 = 33%.
Ebrahimi [8]	≥6 months	-	No significant effect between the two groups on suicidality; no difference by diabetes status, or agent (liraglutide, semaglutide, etc.) either.	N/R	RiskRatio: 0.76; 95% CI, 0.48–1.21; *p* = 0.24; I2 = 0.0%.
Pierret [9]	28 weeks	N/R	No significant results on suicidality (although only three studies assessed suicidality alone and an effect size could not be pooled for it).	No significant effects on serious, non-serious psychiatric disorders, or depression changes. Small improvements in eating restraint (g = 0.35), emotional eating (g = 0.32), and quality of life (mental-health-related, physical-health-related, diabetes-related, weight-related); g ranging from 0.15 to 0.27.	Serious psychiatric AE: logRiskRatio −0.02 (95% CI −0.20 to 0.17); non-serious: logRiskRatio −0.03 (95% CI −0.21 to 0.16); depression: g 0.02 (95% CI −0.51 to 0.55); eating restraint: g 0.35; and emotional eating: g 0.32.
Silverii [17]	91 weeks	N/R	No significant effects on suicidality.	No significant effects on depression and anxiety.	The Mantel–Haenzel odds ratio (MH-OR) was used. MH-OR suicidality 95%, *p* = 0.61, I2 = 0%, MH-OR depression 95%, *p* = 0.82, I2 = 0%, MH-OR anxiety 95%, *p* = 0.66, I2 = 0%.
Strumila [26]	N/A	Potential risks related to rapid weight loss were as follows: allostatic load, catecholamine surge, serotonin restriction, endotoxemia, and psychological risks (unrealistic expectations and identity changes).	No significant effects on suicidality; a causal effect on suicidality was not supported. Bradford Hill criteria (strength of association, consistency, specificity, temporality, biological gradient, plausibility, coherence, experiment, and analogy) were not met. The association was weak, inconsistent, non-specific, and there was no proof related to a possible increase in suicidal ideation/behaviors after increasing the GLP-1RAs dose. However, a potential increase in suicidal ideation/behavior might follow after a failure of treatment meeting personal expectations.	Improvement in quality of life, decreased risk of anxiety disorders, or potential effectiveness on alcohol consumption (evidence in mice studies).	N/A
Valenta [10]	N/R	Some studies reported adverse effects such as depressive and anxious symptoms, while another study found nervousness, stress, eating disorders, insomnia, binge eating, fear of injection, fear of eating, and self-induced vomiting.	There were mixed results, as follows: – Animal/human studies suggest possible protective effects (reduced risk of suicidal ideation via anti-inflammatory/neuroprotective mechanisms). – Pharmacovigilance reports note cases of depression, anxiety, and suicidal ideation, but causality is uncertain.	Potential effectiveness in improving depression and anxiety, cognition, alcohol/substance use, and binge eating. Mostly, there was an improvement trend for depression, cognition, and substance use, binge eating (in both animals and human studies). Mixed effects were found for anxiety (although protective in most contexts, anxiogenic in acute high-dose rodent models). Neutral/minimal effect for psychosis/autism (very limited evidence).	N/A
Valentino [32]	N/R	N/R	For cohort studies, results showed no evidence of an increase in suicidality. However, a reduced risk for suicidality in adolescents with obesity was reported in one study. Mixed results were found in pharmacovigilance, as follows: an increased risk was observed for semaglutide and liraglutide, whereas for the rest of the GLP-1RAs this trend was not supported.	N/R	
Wei 2024 [25]	N/R	N/R	Overall, a non-significant decreased trend in suicidal ideation was found. However, an increase in self-harm in those who were underweight was found to be significant.	N/R	Overall Risk Ratio: 0.83, *p* = 0.06. Risk ratio for underweight patients and self-injury: 1.05, *p* < 0.03.

Note: N/R = not reported; N/A = not applicable; *p* = level of significance; CI = confidence interval; I2 = level of heterogeneity; AE = adverse effects.

**Table 3:** Quality assessment of the included reviews.

Review	Quality
Barroso 2024 [27]	Moderate
Breit 2025 [28]	Moderate
Bushi 2025 [29]	Good
Di Stefano 2025 [30]	Moderate
Ebrahimi 2025 [8]	Good
Pierret 2025 [9]	Moderate
Silverii 2023 [17]	Poor
Valenta 2024 [10]	Moderate
Valentito 2025 [32]	Good

Note: Studies not found in full text were not included in the assessment.

## Data Availability

No new data were created or analyzed in this study.

## Appendix A

For all databases, the following core search string was used in exact words and order:

“(glucagon-like peptide-1 receptor agonist OR GLP-1 receptor agonist) AND (suicide OR self-injury OR self-harm OR suicidal ideation OR suicidal behavior OR suicide attempt OR suicide risk) AND (diabetes) AND (review OR meta-analysis)”.

The searched databases were as follows: MedLine, PubMed, PsychInfo, Web of Science, Science Direct, Sage, Wiley, Scopus, Google Scholar, Proquest, and Cochrane Library.

The search was not restricted to any part of the text. Also, no restrictions to publication dates or language were applied.

### Appendix A.1. Inclusion Criteria

We included studies that

(a) documented the link between GLP-1RAs and suicidality (meaning suicidal ideation and/or suicide/self-harm attempts);

(b) were synthesis studies (systematic reviews, qualitative/narrative reviews, scoping reviews, or meta-analyses);

(c) were performed on adults and/or children;

(d) were based on samples diagnosed with diabetes or obesity;

(e) addressed suicide ideation or behavior either as a main outcome or as a secondary one. No language filter was employed. For studies written in any other language than English, the text was translated into English with the help of ChatGPT [22], then checked for accuracy by one author who is fluent in Spanish (the language the article was written in; this applied only for one study as all the other studies were written in English).

Eligible studies that could not be accessed in full-text form were also included (although they could not be analyzed completely).

### Appendix A.2. Exclusion Criteria

Studies of any design other than a systematic/qualitative review or meta-analysis, as well as studies that did not address suicide ideation or behavior, were excluded.
